# Role of *EZH2* Polymorphisms in Esophageal Squamous Cell Carcinoma Risk in Han Chinese Population

**DOI:** 10.3390/ijms150712688

**Published:** 2014-07-17

**Authors:** Zhen-Bin Ma, Guang-Hong Guo, Qiong Niu, Ning Shi

**Affiliations:** 1 Department of Gastroenterology, Affiliated Hospital of Binzhou Medical College, No. 661, Yellow-River Second Street, Binzhou 256600, Shandong, China; E-Mails: mazhenbin@yeah.net (Z.-B.M.); niuqiong@126.com (Q.N.); shiningbyfy@yeah.net (N.S.); 2 Department of Gynaecology, Affiliated Hospital of Binzhou Medical College, No. 661, Yellow-River Second Street, Binzhou 256600, Shandong, China

**Keywords:** allele, *EZH2*, esophageal squamous cell carcinoma, polymorphism

## Abstract

Gene single nucleotide polymorphisms play a critical role in the development of esophageal squamous cell carcinoma (ESCC). The aim of this study is to investigate the associations between *EZH2* gene polymorphisms and ESCC risk. We undertook a case-control study to analyze three *EZH2* polymorphisms (148505302C > T, 2110 + 6A > C and 626 − 394T > C) in an Han Chinese population, by extraction of genomic DNA from the peripheral blood of 476 patients with ESCC and 492 control participants, and performed *EZH2* genotyping using DNA sequencing. The obtained results indicated that overall, no statistically significant association was observed in 148505302C > T and 2110 + 6A > C. However, 626 − 394T > C genotype was at increased risk of ESCCs (*p* = 0.006; odds ratio (OR) = 1.131, CI 95%: 1.034–1.236). Moreover, 626 − 394C/C genotype ESCCs were more significantly common in patients with tumor size of >5 cm than T allele ESCC and in cases of poor differentiation and lower advanced pathological stage. In conclusion, polymorphism in 626 − 394T > C was observed to be associated with susceptibility of ESCC. Nevertheless, further investigation with a larger sample size is needed to support our results.

## 1. Introduction

Esophageal cancer is an aggressive cancer constituting the sixth cause of cancer-related deaths worldwide [1]. The main histological type is squamous cell carcinoma (SCC) in East Asia [2]. Despite the great advances achieved in operation and chemoradiotherapy technology recently, the overall 5-year survival rate of esophageal squamous cell carcinoma (ESCC) patients remains less than 30% [3,4]. Accumulated epidemiological evidences indicate that tobacco smoking, heavy alcohol drinking, micronutrient deficiency as well as dietary carcinogen exposure might be the main environmental risk-factors of this malignant disease [5]. Many studies have suggested the importance of gene single nucleotide polymorphisms (SNPs) that are involved in xenobiotic metabolism that might be responsible for ESCC risk. A number of common SNPs associated with ESCC risk play a critical role in the development of ESCC through modifying the expression of target genes that regulate cell behaviors [6,7]. Therefore, discovery of novel biologically functional and ESCC-risk-associated SNPs might be a potentially valuable path towards illuminating ESCC genetics thoroughly.

Polycomb group genes (PcGs) are epigenetic effectors essential for stem cell self-renewal and lineage-specific gene silencing [8]. The enhancer of zeste homolog 2 (*EZH2*) gene, known as a member of the PcG, has been found to contribute to the maintenance of cell identity, cell cycle regulation and oncogenesis [9]. It is reported that *EZH2* serves as a histone methyl transferase involved in gene silencing, and disruption of *EZH2* expression can lead to cancer [10]. *EZH2* amplification was first reported in hematologic malignancies [11,12], and there is increasing evidence showing that *EZH2* is frequently overexpressed in a wide variety of cancerous tissue types, including endometrial, gastric, colon, esophageal, hepatocellular, bladder and oral cancers and is associated with poor prognosis [13,14,15,16,17,18].

The abnormalities of *EZH2* were observed to correlate closely with tumor aggressiveness and/or poor patient prognosis. He and colleagues investigated the expression of EZH2 in ESCC by IHC [19]. The authors concluded that high expression of EZH2 correlates with tumor aggressiveness and adverse patient outcome in ESCC treated with definitive chemoradiotherapy. In our previous study, we observed that polymorphism in *EZH2* gene 626 − 394T > C was observed to be associated with susceptibility of colorectal cancer. However, 148505302C > T polymorphism was indicated to play a protective role in susceptibility to colorectal cancer [20].

Thus, in this study, in order to clarify association between *EZH2* SNPs rs887569 (g.148505302C > T), rs41277434 (c.2110 + 6A > C) and rs3757441 (c.626 − 394T > C) polymorphisms and ESCC risks, we performed a hospital-based case-control study on Han Chinese population.

## 2. Results

### 2.1. Characteristics of Subjects

This study comprised 476 patients and 492 controls. All the cases and controls were randomly selected from the general Han Chinese population of China. Table 1 shows the main characteristics of case-control populations. The age distribution, gender, alcohol consumption, smoking habits, and family history (FH) of cancer in the case and control groups are not statistically different. The cases and controls were well matched by age (mean ± SD, 62.36 ± 5.41 years in cases and 63.45 ± 4.18 years in controls) and gender (the same proportion for males and females), which suggests that frequency matching was adequate. The frequency of males was significantly higher, being in accordance with a worldwide estimation for ESCC.

**Table 1:** General characteristics for the esophageal squamous cell carcinoma (ESCC) cases and control population.

Parameters	No. of Cases (%) *N* = 476	No. of Controls (%) *N* = 492	*p* Value ^c^
Age ^a,b^			
≤45	38 (8.0)	43 (8.7)	0.818
45–69	349 (73.3)	352 (71.5)	
≥70	89 (18.7)	97 (19.7)	
Sex			
Female	135 (28.4)	154 (31.3)	0.318
Male	341 (71.6)	338 (68.7)	
Alcohol drinking			
No	165 (34.7)	187 (38.0)	0.280
Yes	311 (65.3)	305 (62.0)	
Cigarette smoking			
No	227 (47.7)	234 (47.6)	0.968
Yes	249 (52.3)	258 (52.4)	
Family history of cancer			
No	385 (80.9)	390 (79.3)	0.530
Yes	91 (19.1)	102 (20.7)	

^a^ Age of diagnosis for cases; ^b^ Age of control population at the time of diagnosis for the matched case; ^c^*p* value obtained by χ^2^ (cases *vs.* control group).

### 2.2. EZH2 Gene 148505302C > T, 2110 + 6A > C and 626 − 394T > C Polymorphisms in ESCC

The gene polymorphisms of *EZH2* rs887569 (g.148505302C > T), rs41277434 (c.2110 + 6A > C) and rs3757441 (c.626 − 394T > C) were successfully amplified in all ESCC and control cases. The genotypic distributions of all three gene polymorphisms in cases and controls were in Hardy-Weinberg equilibrium (all *p* > 0.05) (Table 2). Overall, no statistically significant association was observed in *EZH2* SNP 2110 + 6A > C and 148505302C > T. Individuals with *EZH2* 626 − 394C/C genotype were more susceptible to ESCCs (*p* = 0.004, OR = 1.324). Moreover, the variant allele frequency C of *EZH2* (626 − 394T > C) was higher in cases as compared with controls (24.2% *vs.* 21.8%); this result also showed statistical significance (*p* = 0.006).

**Table 2:** Association between *EZH2* gene 148505302C > T, 2110 + 6A > C and 626 − 394T > C polymorphisms and ESCCs.

Genotype	Cases ^a^, *n* (%) (*N* = 476)	Controls ^a^, *n* (%) (*N* = 492)	*p* Value	Crude OR (95% CI)	Adjusted OR (95% CI) ^b^	
148505302C > T						
CC	126 (26.5)	129 (26.2)		1 (Reference)	1 (Reference)	
CT	253 (53.2)	264 (53.7)	0.901	0.991 (0.855–1.148)	0.997 (0.867–1.108)	
TT	97 (20.3)	99 (20.1)	0.987	1.002 (0.833–1.204)	1.028 (0.734–1.455)	
C allele	505 (26.1)	522 (27.0)				
T allele	447 (23.1)	462 (23.9)	0.999	1.000 (0.916–1.092)		
2110 + 6A > C						
AA	133 (27.9)	141 (28.7)		1 (Reference)	1 (Reference)	
AC	242 (50.8)	231 (46.9)	0.490	1.054 (0.909–1.221)	1.067 (0.914–1.432)	
CC	101 (21.3)	120 (24.4)	0.529	0.948 (0.802–1.120)	0.971 (0.889–1.257)	
A allele	508 (26.2)	513 (26.5)				
C allele	444 (22.9)	471 (24.3)	0.589	0.976 (0.894–1.066)		
626 − 394T > C						
TT	112 (23.5)	147 (29.9)		1 (Reference)		1 (Reference)
CT	260 (54.6)	267 (54.3)	0.108	1.120 (0.978–1.283)		1.135 (0.942–1.341)
CC	104 (21.9)	78 (15.8)	0.004	1.324 (1.086–1.615)		1.436 (1.286–1.812)
T allele	484 (25.0)	561 (29.0)				
C allele	468 (24.2)	423 (21.8)	0.006	1.131 (1.034–1.236)		

^a^ The χ^2^ for HWE of *EZH2* gene 148505302C > T, 2110 + 6A > C and 626 − 394C > T polymorphisms in case and control group is 2.14 and 2.93, 0.22 and 1.73, and 4.09 and 5.65 respectively (all *p* > 0.05); ^b^ ORs were adjusted for gender, age (≤45, 45–69 and ≥70 years), smoking status, alcohol consumption and family history (FH) of cancer.

### 2.3. Relationship between EZH2 Gene 148505302C > T, 2110 + 6A > C and 626 − 394T > C Polymorphism and Known Clinicopathological Variables

Table 3 shows the association of *EZH2* gene 148505302C > T, 2110 + 6A > C and 626 − 394T > C polymorphisms with clinicopathological characteristics, including gender, age at diagnosis, tumor size, differentiation, T stage, lymph node metastasis, and pathological stage of the cancer.

For all three SNPs, the polymorphisms were not related to the gender and age of the patients and pathological features of the cancer (*p* > 0.05). Our data indicated that *EZH2* gene (626 − 394T > C) polymorphism may be a susceptible genotype for ESCC development and may increase the risk of ESCC among the Han Chinese population. Moreover, 626 − 394T > C SNP was observed to be significantly associated with increased risk with tumor size, differentiation, T stage, and pathological stage (*p* = 0.014, *p* = 0.001, *p* = 0.017 and *p* = 0.037, respectively). However, the 148505302C > T and 2110 + 6A > C polymorphism in *EZH2* gene may be not association with ESCC susceptibility.

Although in *EZH2* gene 626 − 394T > C the polymorphisms were not related to the presence of lymph nodal metastases, the 626 − 394CC genotype was more common in ESCC of higher advanced pathological staging (stages I & II *vs.* III & IV, *p* = 0.037).

**Table 3:** Clinicopathological relevance of *EZH2* gene 148505302C > T, 2110 + 6A > C and 626 − 394T > C polymorphisms in ESCC.

Parameters	148505302C > T		*p* Value	2110 + 6A > C		*p* Value	626 − 394T > C		*p* Value
	CC + CT (%)	TT (%)		AA + AC (%)	CC (%)		TT + TC (%)	CC (%)	
Gender									
Female	102 (21.4)	33 (6.9)	0.166	105 (22.1)	30 (6.3)	0.736	104 (21.8)	31 (6.5)	0.711
Male	277 (58.2)	64 (13.4)		270 (56.7)	71 (14.9)		268 (56.3)	73 (15.3)	
Age									
<55 years	174 (36.6)	42 (8.8)	0.645	175 (36.8)	41 (8.6)	0.277	173 (36.3)	43 (9.0)	0.350
≥55 years	205 (43.1)	55 (11.6)		200 (42.0)	60 (12.6)		199 (41.8)	61 (12.8)	
Size									
≤5 cm	175 (36.8)	45 (9.5)	0.969	178 (37.4)	42 (8.8)	0.293	183 (38.4)	37 (7.8)	0.014
>5 cm	204 (42.9)	52 (10.9)		197 (41.4)	59 (12.4)		189 (39.7)	67 (14.1)	
Differentiation									
Good	85 (17.9)	26 (5.5)	0.313	91 (19.1)	20 (4.2)	0.223	98 (20.6)	13 (2.7)	0.001
Moderate	121 (25.4)	35 (7.4)		127 (26.7)	29 (6.1)		127 (26.7)	29 (6.1)	
Poor	173 (36.3)	36 (7.6)		157 (33.0)	52 (10.9)		147 (30.9)	62 (13.0)	
T stage									
1 + 2	192 (40.3)	54 (11.3)	0.378	187 (39.3)	59 (12.4)	0.127	203 (42.6)	43 (9.0)	0.017
3 + 4	187 (39.3)	43 (9.0)		188 (39.5)	42 (8.8)		169 (35.5)	61 (12.8)	
Lymph node metastasis									
Present	203 (42.6)	57 (12.0)	0.359	205 (43.1)	55 (11.6)	0.970	205 (43.1)	55 (11.6)	0.687
Absent	176 (37.0)	40 (8.4)		170 (35.7)	46 (9.7)		167 (35.1)	49 (10.3)	
TNM pathological stage									
I and II	181 (38.0)	45 (9.5)	0.810	176 (37.0)	50 (10.5)	0.646	186 (39.1)	40 (8.4)	0.037
III and IV	198 (41.6)	52 (10.9)		199 (41.8)	51 (10.7)		186 (39.1)	64 (13.4)	

## 3. Discussion

Polycomb group (PcG) proteins are epigenetic chromatin modifiers involved in cancer development and their roles are now being evaluated in numerous human malignancies [21]. PcG proteins are essential for cancer stem cell (CSC) self-renewal. PcG members are organized in two main protein complexes: Polycomb repressive complex 1 and 2 (PRC1, PRC2) [22]. Enhancer of zeste homolog 2 (EZH2) is the catalytic subunit of PRC2, and is overexpressed in poorly differentiated ESCC [19]. Several genetic and epigenetic factors may be involved in the deregulation and modulation of key signaling pathways in tumor aggressiveness and chemoresistance. The abnormal expression of EZH2 is involved in the tumorigenic types of cancer with poor prognoses [18,23]. Previous studies have demonstrated that EZH2 contributes to the epigenetic silencing of several target genes that control cell growth and proliferation, including E-cadherin, Rb and p16 [24]. The overexpression of EZH2 may induce hypermethylation of the promoter of the p16 gene, reducing the expression of p16, which is a key step in the multistep cholangiocarcinogenesis from hepatolithiasis to intraepithelial neoplasia [25].

In one of our previous studies, we undertook a case-control study to analyze associations between *EZH2* polymorphisms (148505302C > T, 2110 + 6A > C and 626 − 394T > C) and colorectal cancer (CRC) risk [20]. Our results indicated that no statistically significant association was observed in 2110 + 6A > C SNP. Nevertheless, 148505302C > T genotype demonstrated a protective effect in CRCs (odds ratio (OR) = 0.777). Furthermore, 148505302 T allele CRC was more significantly common in patients with smaller tumor size, good differentiation and lower advanced pathological stage. However, 626 − 394T > C genotype CRCs were more significantly common in patients with tumor size of >5 cm than T allele CRC and in cases of poor differentiation and lower advanced pathological stage.

The present study shows that there is no association between the *EZH2* gene 148505302C > T SNP, 2110 + 6A > C SNP and ESCCs development, which might indicate genetic diversity linked with tumorigenesis. In another previous study, Crea and colleagues have shown that the haplotypes analysis of 2110 + 6A > C genotype was not related with objective response, progression-free survival (PFS) and overall survival (OS) in metastatic colorectal cancer patients (mESCC) [26]. Although the present study did not reveal that the *EZH2* g.148505302 T allele has a significant relationship with ESCC risk, Paolicchi and colleagues reported that *EZH2* g.148505302 TT genotype was correlated with a significantly longer OS in cholangiocarcinoma (*p* = 0.026), and moreover, the TT genotype revealed a trend toward a significant association with a reduced risk of mortality [27]. Therefore, the association of *EZH2* g.148505302C > T and 2110 + 6A > C polymorphism with susceptibility of ESCCs should be studied in a larger sample size, and the involved molecular mechanisms need further investigation.

The 626 − 394T > C SNP is an intronic polymorphism [27]. This intronic SNPs may affect gene expression through several mechanisms, including changes in transcription factor (TF) binding sites [28], microRNA target sequences [29] and splicing variants [30]. In C/C homozygotes, EZH2 expression could be deregulated, therefore producing more malignant tumors. Crea found that *EZH2* mRNA levels are higher in C/C CRC patients, compared with other genotypes. The C/T and T/T genotypes did not show a significantly different EZH2 expression [26]. In a recent study, Fornaro *et al.* revealed that *EZH2* gene 626 − 394C > T SNP does not represent a potential predictive marker for bevacizumab efficacy but may play a role as either a prognostic variable or a predictive factor for first-line irinotecan-based chemotherapy in CRC patients [31]. In this study, our data for 626 − 394T > C variant showed that the C allele distribution was significantly associated with ESCCs, compared with controls (*p* = 0.006). The homozygous CC variant in our study was observed to be significantly associated with increased risk of tumor size, differentiation, T stage, and pathological stage.

## 4. Materials and Methods

### 4.1. Subjects

A total of 476 cases of patients with ESCC and 492 healthy controls were qualified for this study. We performed a hospital-based case-control study. All samples were collected before any kind of therapeutic measures were taken at the Department of Gastroenterology, Affiliated Hospital of Binzhou Medical College, between January 2009 and October 2013. The samples of the patients were collected after the diagnosis had been confirmed by esophageal endoscopy. All patients were submitted to surgery or preoperative chemoradiotherapy. Written informed consent was obtained from all participants. The study protocol was approved by the Ethics Committee of the Affiliated Hospital of Binzhou Medical College in accordance with the Declaration of Helsinki (2000). The ESCC patients were staged according to the American Joint Committee on Cancer/International Union Against Cancer tumor-node-metastasis (TNM) staging system [32].

### 4.2. DNA Extraction

Genomic DNA from the whole blood cells was extracted using a QIAamp Blood kit (Qiagen, Hilden, Germany), following the manufacturer’s instructions. DNA concentration and purity of each sample were measured by ultraviolet spectrophotometer (Eppendorf, Hamburg, Germany). DNA samples were routinely stored at −20 °C.

### 4.3. Genotyping

Analysis of *EZH2* gene SNPs, rs887569 (g.148505302C > T), rs41277434 (c.2110 + 6A > C) and rs3757441 (c.626 − 394T > C) were performed using multiplex polymerase chain reaction (PCR) with an ABI premix. Primers for PCR and single base extension were designed using the Assay Designer software package (Sequenom, San Diego, CA, USA). In each 25 μL reaction, 1 μL genomic DNA (100 ng/μL) was amplified by 1.25 U Taq DNA polymerase (Takara, Dalian, China) with 2 μL of 2.5 mM dNTPs and 0.5 μL of each primer. The PCR conditions were performed as previously described [20,33]. PCR products were analyzed on a 3% ethidium bromide added agarose gel, and photographs were taken under ultraviolet light transilluminator. Subsequently, PCR product was sequenced in an ABI PRISM 3100 sequencer (Applied Biosystems, Milton Freewater, CA, USA) using BigDye Terminator v3.1 (Applied Biosystems, Milton Freewater, CA, USA) Cycle Sequencing method (Applied Biosystems, Milton Freewater, CA, USA) as recommended by the manufacturer. Candidate SNP regions were detected and typed with the aid of DNA Star Software (DNASTAR, Madison, WI, USA).

### 4.4. Statistical Analysis

Statistical calculations were performed using the SPSS Statistics 13.0 for Windows software package (SPSS Inc., Chicago, IL, USA). Frequency and susceptibilities of mutations were compared with the χ^2^ test. The *p* values obtained were two-tailed, and the association of significance was assumed to be less than 0.05. The Hardy-Weinberg equilibrium (HWE) was verified for the different studies of polymorphisms. *p* > 0.05 was considered not to deviate from the equilibrium. The crude and adjusted odds ratio (OR) and the corresponding 95% confidence intervals (CI) were calculated using unconditional multiple logistic regression.

## 5. Conclusions

To conclude, this is the first experience suggesting that an *EZH2* polymorphism has significant impact on clinical outcome in ESCC. Despite this, our investigation has some limitations that should be pointed out: it is a retrospective study, conducted on a small population. Thus, our exploratory data need to be confirmed by a larger, prospective independent series in order to overcome possible bias inherent to retrospective evaluations. In particular, to explore the predictive value of this SNP, an adequately powered, prospective randomized trial should be carried out.
